# Real-time, automatic, open-source﻿﻿ sleep stage classification system using single EEG for mice

**DOI:** 10.1038/s41598-021-90332-1

**Published:** 2021-05-27

**Authors:** Taro Tezuka, Deependra Kumar, Sima Singh, Iyo Koyanagi, Toshie Naoi, Masanori Sakaguchi

**Affiliations:** 1 Faculty of Library, Information and Media Science/Center for Artificial Intelligence Research (C-AIR), University of Tsukuba, Tsukuba, Japan; 2 International Institute for Integrative Sleep Medicine (WPI-IIIS), University of Tsukuba, Tsukuba, Japan

**Keywords:** REM sleep, Sleep, Software, Electroencephalography - EEG, Single-channel recording, Software, Information technology, Computer science

## Abstract

We developed a real-time sleep stage classification system with a convolutional neural network using only a one-channel electro-encephalogram source from mice and universally available features in any time-series data: raw signal, spectrum, and zeitgeber time. To accommodate historical information from each subject, we included a long short-term memory recurrent neural network in combination with the universal features. The resulting system (UTSN-L) achieved 90% overall accuracy and 81% multi-class Matthews Correlation Coefficient, with particularly high-quality judgements for rapid eye movement sleep (91% sensitivity and 98% specificity). This system can enable automatic real-time interventions during rapid eye movement sleep, which has been difficult due to its relatively low abundance and short duration. Further, it eliminates the need for ordinal pre-calibration, electromyogram recording, and manual classification and thus is scalable. The code is open-source with a graphical user interface and closed feedback loop capability, making it easily adaptable to a wide variety of end-user needs. By allowing large-scale, automatic, and real-time sleep stage-specific interventions, this system can aid further investigations of the functions of sleep and the development of new therapeutic strategies for sleep-related disorders.

## Introduction

The function of sleep has historically been investigated using sleep deprivation. Although this technique reveals the importance of sleep, it is difficult to establish causal relationships between sleep and observed phenotypes due to confounding factors accompanying sleep deprivation (e.g., stress). To avoid this issue, genetic methods were introduced to manipulate neural circuits that regulate sleep. However, these neural circuits are often hard-wired to those vital for other physiological processes (e.g., orexin neurons to appetite, melanin-concentrating hormone neurons to memory). Recently, optogenetics has revolutionized investigation into the function of sleep. Using optogenetics, target neuronal circuits can be specifically manipulated with light to interrogate their causal contributions during specific sleep stages while leaving sleep structure unchanged^1–3^. When using this technique, it is essential to classify a subject’s sleep stage in real-time for interrogation. However, a real-time, automatic, and scalable system for sleep stage classification for mice is lacking. Mice show a clear distinction between different sleep stages (i.e., rapid eye movement sleep (REM) and non-REM sleep (NREM)) and permit advanced genetic interventions. However, specifically examining the function of REM is difficult, mainly owing to its short and sparse nature (i.e., average ~ 1 min per episode, ~ fivefold less than NREM). Therefore, real-time, sensitive, and specific detection of REM in mice remains a challenge.

Real-time classification carries additional difficulties compared with offline classification, as parameters must be adjusted automatically for each subject, and artifacts and noise should be processed in real time. For example, Izawa et al. used a rule-based, real-time mouse sleep stage classification system^2^ in which thresholds are manually calibrated for each mouse before starting real-time classification, making it ill served for large-scale application. Furthermore, waveform patterns characteristic of each sleep stage (e.g., slow-wave activity, theta oscillations) are often defined by spectrum features using Fast Fourier Transformation (FFT). Indeed, Patanaik et al. developed a real-time human sleep classification system with a convolutional neural network (CNN) using FFT, but not raw, data from two-channel electroencephalogram (EEG) and two-channel electrooculogram^4^. They achieved 81.4% overall accuracy (ACC), 71.8% REM sensitivity, and 83.6% REM specificity. However, transformation of EEG waveform data to spectrum (i.e., FFT) data may lose transient wave characteristics that are potentially useful for stage classification. Most recently, Garcia-Molina et al. developed a system that classifies human sleep in real-time using one-channel EEG (1EEG)^5^. This system processes raw signals using a CNN with a long short-term memory network (LSTM) to incorporate historical information, which achieved Cohen's kappa values very close to those of professional judges and 72–94% REM specificity. However, a system that can robustly and automatically classify sleep stages in real time is still lacking.

Here, we report two fully automatic, real-time sleep stage classification systems for mice using 1EEG that use features common to any time-series data, which is unprecedented in existing systems. Our method is scalable and easily harnessed in any closed feedback loop system for real-time intervention in specific sleep stages, with especially high sensitivity and specificity for REM.

## Results

### Development of a sleep stage classification system using universal time-series features

**Figure 1:**
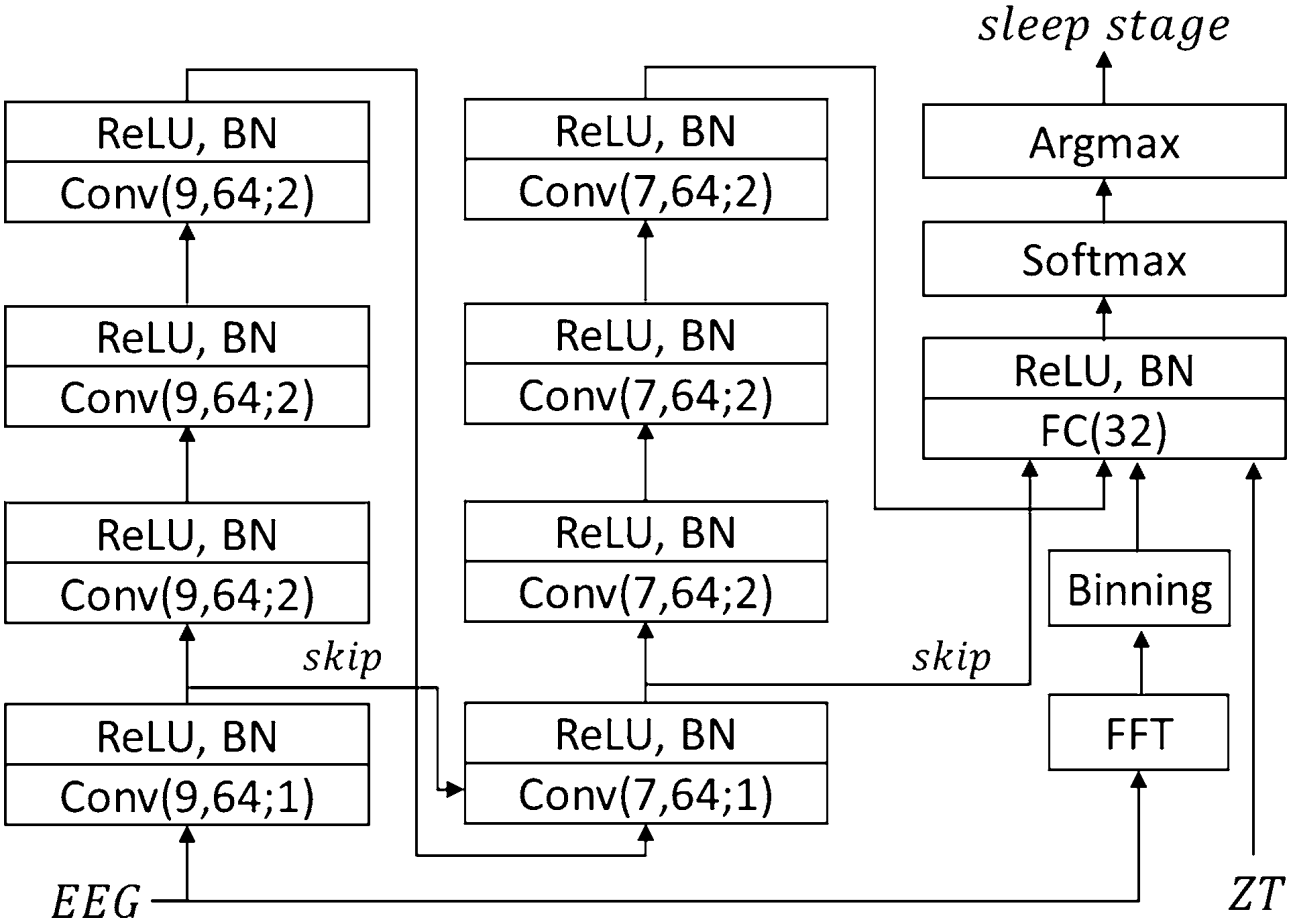
Network structure of the UTSN. Conv($a$,${ }b$;${ }c$) indicates a block consisting of a one-dimensional convolution layer with kernel size $a$ having $b$ channels and stride set to $c$. FC($d$) is a block composed of a fully connected layer having $d$ output channels. FFT, Fast Fourier Transformation; skip, skip-connection; ReLU, rectified linear unit; BN, batch normalization.

First, we developed a real-time sleep stage classification system with a CNN using 1EEG data from mice, named the universal time-series network (UTSN). UTSN processes raw EEG, FFT, and zeitgeber time (ZT) together. ZT is used because sleep is regulated by circadian rhythm^6^. The output from the CNN, FFT, and ZT are concatenated and transformed into a three-dimensional vector corresponding to the probabilities of each sleep stage (i.e., wakefulness, NREM, and REM) by a fully connected neural network (FCN) (Fig. 1). We did not use any de-noising preprocessor for the raw EEG signals, as the multi-layer architecture of the CNN can act as a set of filters and extract only meaningful information for sleep stage classification^7^.

To make the UTSN applicable to closed feedback loop applications, we set the epoch window to 10 s, which was shown to be useful for optogenetic manipulation during REM^3^. For the activation function to nonlinearly transform the signal, we used a rectified linear unit (ReLU). Each convolution layer is followed by a ReLU activation layer and subsequent batch normalization (BN). BN standardizes the activity of each layer and prevents the vanishing gradient problem that occurs in deep learning. Two skip-connections^8^ are inserted to capture both crude and detailed characteristics. The 1–12 Hz range of the FFT spectrum, which covers the critical characteristic oscillatory activity in delta (1–4 Hz) and theta (6–9 Hz) bands, was split into 22 bins (i.e., 22-dimensional feature vector, 0.5 Hz per bin). We also implemented classifiers that use the short-time Fourier transform (STFT) instead of FFT, which resulted in marginal improvement (Suppl. Figs. 1–4, Suppl. Tables 5–7). As the task is multiclass classification, the softmax function converts the output of the FCN to a vector representing a probability distribution over sleep stages. Finally, the argmax function outputs the sleep stage with the highest estimated probability.

### Addition of LSTM

**Figure 2:**
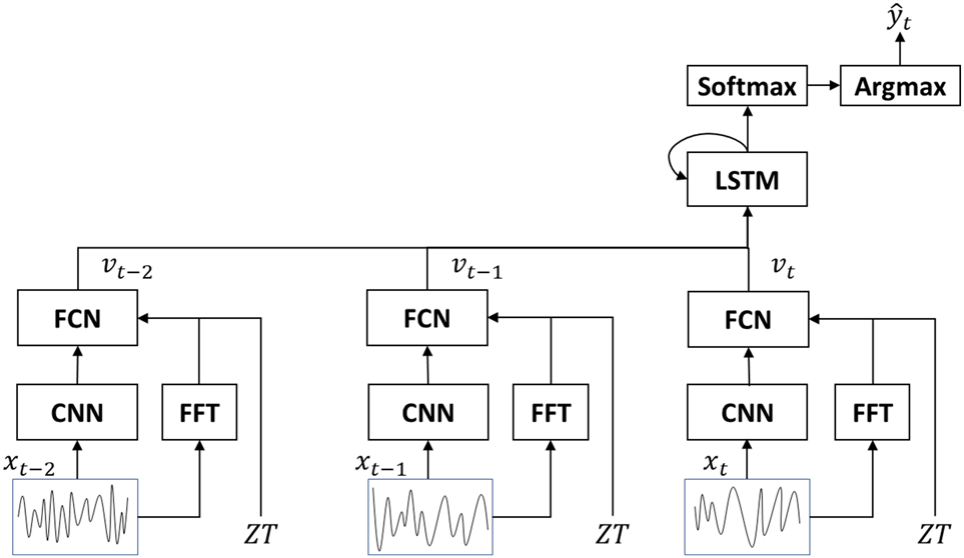
UTSN-L architecture. The signal from each epoch is processed using the same architecture as the UTSN until the FCN (i.e., before the final softmax function). $x_{t}$, $x_{t - 1}$, … $x_{t - m + 1}$ are EEG signals for epochs $t$ to $t - m + 1$, respectively. The output of the FCN is a sequence of low dimensional feature vectors, $v_{t}$, $v_{t - 1}$, … $v_{t - m + 1}$, which is sent to the LSTM. $\hat{y}_{t}$ is the final output that predicts the sleep stage for epoch $t$. The diagram shows a case in which $m = 3$ ($m = 10$ in the real implementation).

We hypothesized that historical information would be useful for classification, as previously shown^5,7^. Therefore, we developed another system (UTSN-L) that connects to a LSTM after FCN output from the UTSN (Fig. 2), which enables the use of the output layer (i.e., before the softmax function) of the UTSN. LSTM is a sequence processing neural network that incorporates past states (i.e., epochs) into the classification of present states.

### Training, validation, and testing

We prepared 214 recordings (6 h per recording) with ground truth (i.e., human-scored) tags annotated by human experts. Of these, 192 recordings were used for training and validation of the systems, and 22 were used for testing. For each of the three sets of training and validation sessions, the 192 recordings were randomly split into a training dataset of 182 recordings and a validation dataset of 10 recordings. Thus, we obtained three classifiers (i.e., trained system) for the UTSN, UTSN-L, and other methods for comparison.

### Evaluation of sleep stage classification

We evaluated the performance of the UTSN and UTSN-L for the three trained classifiers by comparison with conventional methods and network models that partially utilize universal features (Table 1). As simple conventional methods, we used logistic regression^5,9^, which usually performs well for small datasets, and random forest^10–12^ and AdaBoost^13,14^, which are ensemble-based methods that perform well for large datasets. Random forest is an ensemble method that combines the results of training numerous decision trees, and AdaBoost is an adaptive variant of boosting that aggregates outputs of weak learners. To evaluate the contribution of each universal feature, we compared simpler CNN models using each feature (i.e., raw EEG, FFT, or ZT) alone or in combination. Each recording consisted of > fivefold fewer REM epochs than NREM epochs or wakefulness. In this case, multiclass statistics may not reflect actual performance, especially for a class with low frequency (i.e., REM). Therefore, for evaluation criteria, we also used specificity, sensitivity, accuracy, precision, F1 score, and Matthews Correlation Coefficient (MCC). Overall, the UTSN-L performed better than the UTSN (Table 1). Although our system is capable of making predictions in real time, all experiments were conducted using already acquired, human-scored data to allow cross-comparisons among methods. To simulate real-time prediction conditions, the classifiers were not allowed to use future data to judge the target epoch. Moreover, as UTSN-L cannot make predictions unless 10 successive epochs are stored, we omitted the prediction results for the first nine epochs from all methods.

**Table 1:** Prediction performance from validation*.*

	UTSN	UTSN-L	LR	RF	AB	Raw	Spec	Raw + Spec	Raw + ZT	Spec + ZT
Overall accuracy (ACC)	88.1	**89.5**	73.3	77.6	77.9	87.9	77.4	87.9	88.4	77.7
multiclass MCC (mMCC)	78.6	**80.7**	49.5	59	59.3	78.2	59.4	78.6	79.1	60.2
**W**										
Sensitivity	83.6	**87.1**	61.3	72.9	72.9	85.9	76.9	84.6	86.5	77.0
Specificity	92.4	**92.9**	82.6	82.0	83.3	90.3	79.5	91.8	91.0	80.0
Accuracy	89.0	**91.1**	74.3	77.9	79.0	88.7	77.9	89.1	89.3	78.2
Precision	87.0	**88.6**	69.8	71.8	72.3	84.9	72.2	86.0	85.6	72.7
F1 score	84.0	**87.3**	63.0	70.3	71.2	84.0	72.2	83.8	85.2	72.5
MCC	76.7	**80.6**	45.0	55.1	55.8	76.2	56.1	76.5	77.3	56.8
**N**										
Sensitivity	**91.4**	90.6	85.7	86.7	84.9	89.2	82.8	90.2	89.7	82.9
Specificity	87.4	89.7	66.4	76.5	78.4	89.5	80.7	89.9	**90.1**	80.9
Accuracy	89.4	**90.4**	76.9	81.5	81.7	89.3	81.2	90.0	89.8	81.4
Precision	90.9	**92.5**	75.6	82.9	83.9	92.1	83.0	92.0	92.4	83.1
F1 score	90.4	**91.2**	79.2	83.2	83.2	89.8	81.2	90.6	90.3	81.3
MCC	78.8	**80.2**	51.2	62.5	61.6	78.4	62.1	78.6	79.4	62.5
**R**										
Sensitivity	87.4	**90.8**	34.8	30.5	48.3	86.6	39.7	86.2	88.1	42.6
Specificity	98.3	98.1	99.1	**99.7**	98.0	98.4	99.2	97.2	98.2	99.0
Accuracy	**97.7**	97.6	95.4	95.7	95.1	**97.7**	95.8	96.6	97.6	95.8
Precision	79.2	75.2	68.2	**87.9**	62.8	79.2	75.9	78.6	77.9	77.7
F1 score	**82.3**	81.9	41.4	42.1	50.5	81.8	47.1	80.3	81.7	49.5
MCC	**81.9**	81.4	44.2	47.7	50.1	81.3	49.8	80.0	81.6	52.0

Average scores from the three sets of training and validation sessions. *LR* logistic regression, *RF* random forest (64 estimators), *AB* AdaBoost (64 estimators), *Raw* raw signal, *Spec* spectrum, *W* wakefulness, *N* NREM, *R* REM. The best result for each criterion is indicated in bold. For details, see Suppl. Fig. 5.

**Figure 3:**
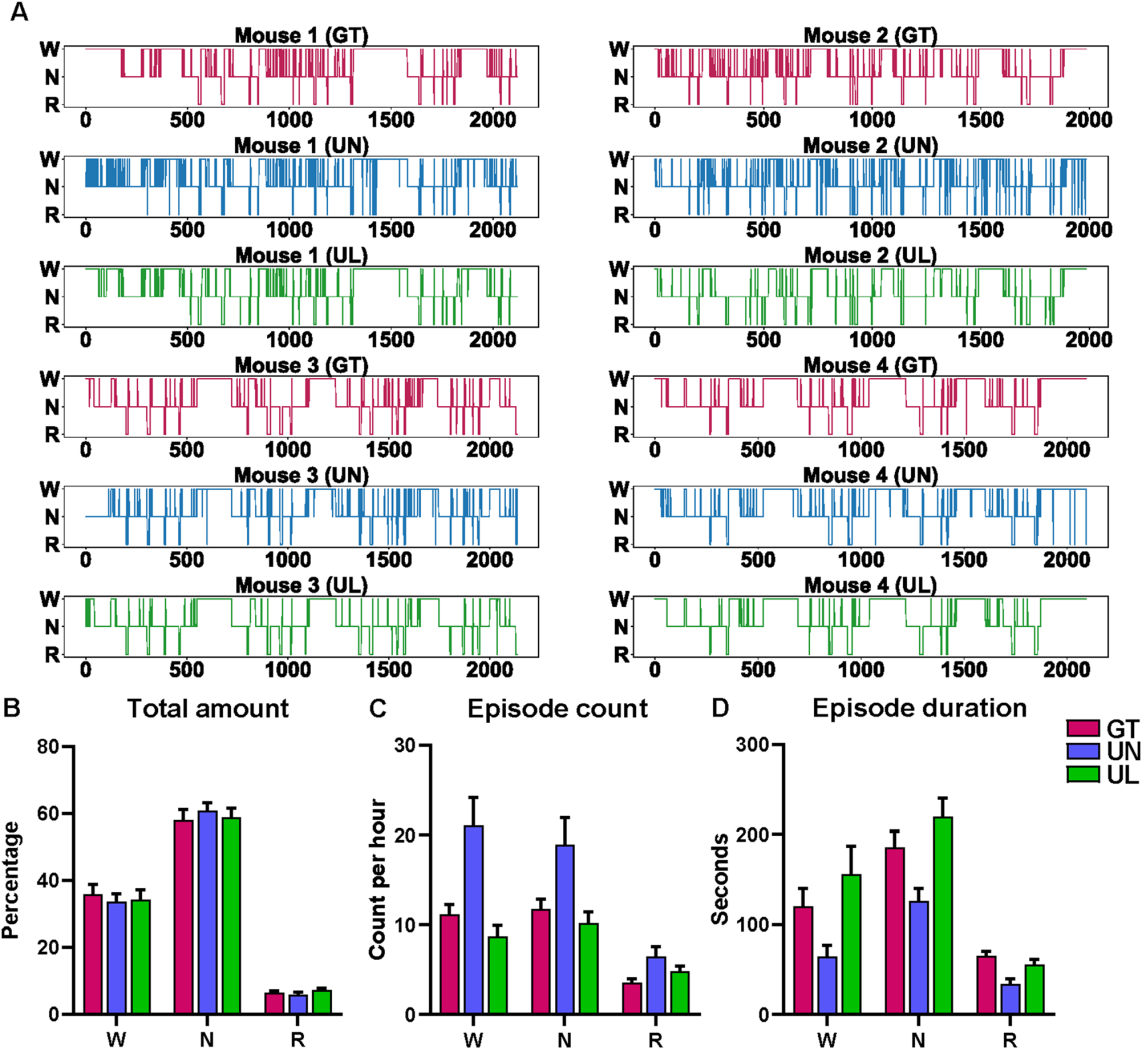
Sleep architecture analysis from testing. (**A**) Hypnogram, (**B**–**D**) sleep architecture. W, wakefulness; N, NREM; R, REM; GT, ground truth; UN, UTSN; UL, UTSN-L; WN, wakefulness to NREM; WR, wakefulness to REM; SN, NREM to wakefulness; NR, NREM to REM; RW, REM to wakefulness; RN, REM to NREM. Data are shown as mean 95% confidence interval (n = 22 recordings).

**Figure 4:**
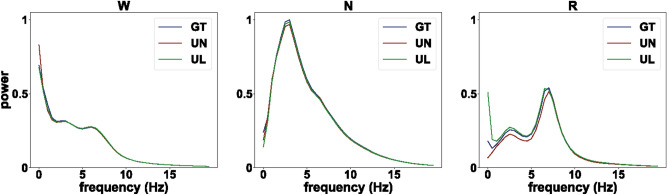
Power spectrum analysis from testing. Power was normalized by the total power (0.5–20 Hz) for each stage. Note the clear peaks within delta range (1–4 Hz) in NREM and theta range (6–9 Hz) in REM. W, wakefulness; N, NREM; R, REM; GT, ground truth; UN, UTSN; UL, UTSN-L; average from n = 22 recordings.

For further evaluation, we chose one trained classifier each for the UTSN and UTSN-L for testing based on ACC score. Overall, the test results were consistent with the validation results (Table 2, Suppl. Table 1, *n* = 22 recordings), which was also supported by tenfold cross-validation (Suppl. Figs. 1–4, Suppl. Tables 5–7). Sleep architectures showed similar results as ground truth (Fig. 3A). Although total sleep amounts were almost identical (Fig. 3B), the UTSN tended to detect more transitions between sleep stages as evidenced by more (Fig. 3C) and shorter (Fig. 3D) episodes. Power spectrum analysis showed almost identical results (Fig. 4), corroborating the waveform patterns of the identified epochs (examples in Suppl. Fig. 6 and VIDEO S1). In summary, these results indicate that including the LSTM together with the three universal features improves the quality of sleep stage classification.

**Table 2:** Confusion matrix analysis from testing.

	$W$	$N$	$R$
**UTSN prediction**			
*Ground truth*			
$W$	9828	1801	104
$N$	1281	25,756	98
$R$	319	368	2265
**UTSN-L prediction**			
*Ground truth*			
$W$	10,008	1585	140
$N$	980	25,718	437
$R$	184	119	2,649

Values, total epoch count from 22 recordings; Shaded cells, true positive; *W* wakefulness, *N* NREM, *R* REM.

### Implementation of a GUI

**Figure 5:**
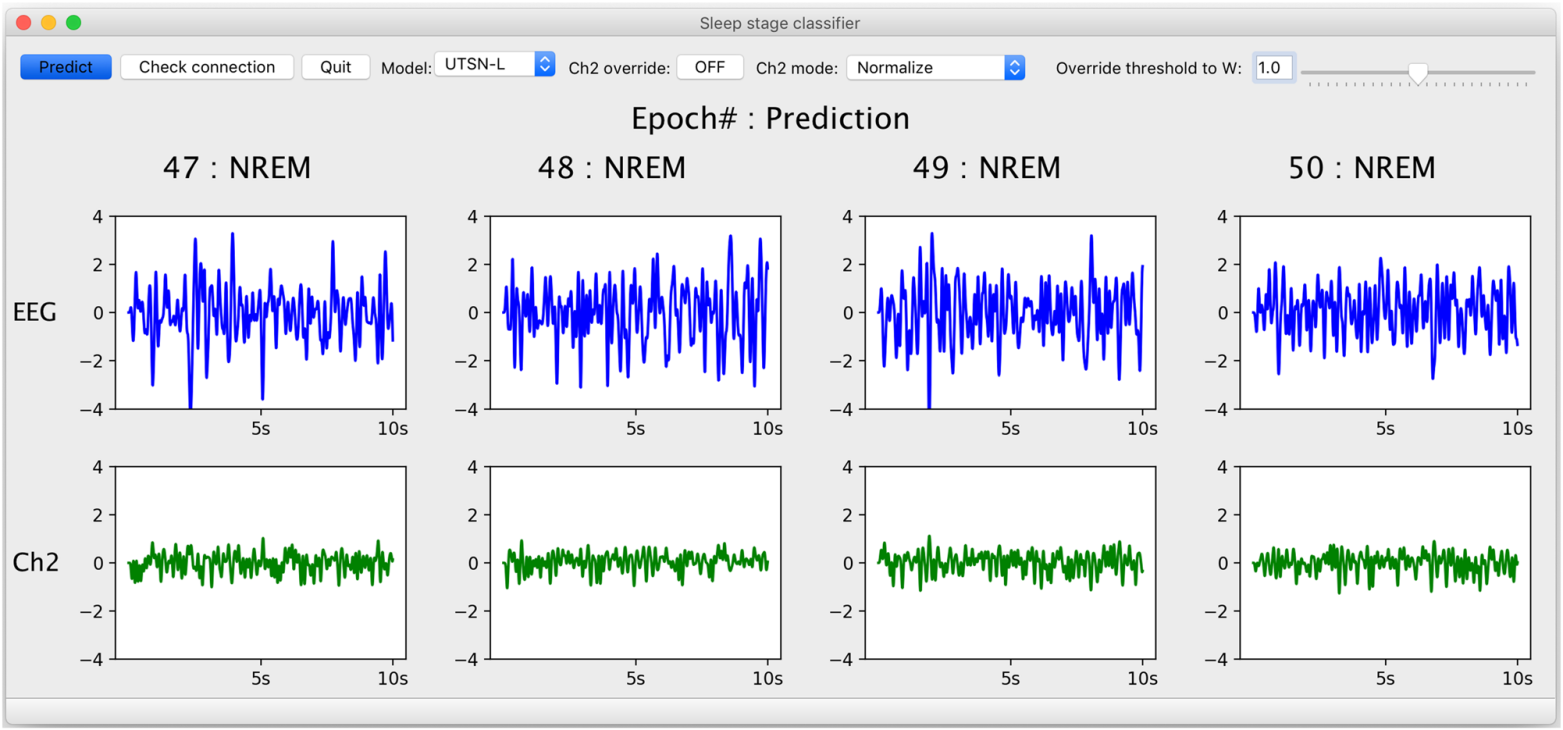
The GUI. Boxes in the upper row show normalized EEG raw signals (blue). Boxes in the bottom row show signals from an open channel (Ch2, green), which can be used for electromyogram (EMG), a motion sensor, an override signal, etc. Predicted stage for each epoch is presented above the corresponding EEG signal. Numbers with bold letters (i.e., 47–50) indicate past epochs (e.g., 10 s each) analyzed by the chosen network. In this case, UTSN-L analyzed epochs 1–50 and is currently analyzing epoch 51 (not predicted at the present moment). As an additional function, users can override the prediction to wakefulness (“W”) if the Ch2 signal crosses a threshold defined by the user, and the threshold for override can be changed manually using a scroll bar in the top-right corner (“Override threshold to W”). Users can also choose whether to normalize the Ch2 signal (“Ch2 mode”). Please see VIDEO S1.

For practical use in experimental protocols without requiring programing skills, we developed a graphical user interface (GUI) that can run on any personal computer capable of running Python and a USB-based data acquisition system (Fig. 5, VIDEO S1). Using this GUI, classifications can also be performed offline using text-based signal data. As the GUI can output classification results in real-time for an Arduino device, it can be immediately implemented in a closed feedback loop system of choice (delay is 10 s due to the epoch window size). The source code is publicly available and thus can easily be customized for specific end-user needs.

## Discussion

Here, we report two real-time, fully automatic mouse sleep stage classification systems using only 1EEG. These systems achieved high sensitivity and specificity for REM and are robust, scalable, and adaptable. They use a 10-s time window, which allows them to be implemented in a closed feedback loop system for functional analysis during specific sleep stages^3^. The use of three universal time-series features allow the systems to be adapted for more detailed classification of sleep stages (e.g., high-theta and low-theta wakefulness^15^) from a variety of signal sources, even those from other species (e.g., humans) and brain areas (e.g., local field potentials in the hippocampus).

We believe that eliminating electromyogram (EMG) from the input markedly expands the system’s applications, particularly when they are applied to species in which reliable EMG is not easily obtained (e.g., reptiles^16^). Moreover, reducing the number of input channels bestows these systems with simplicity and thus scalability against a backdrop of reduced information. Indeed, although there have been attempts to use 1EEG for real-time classification^5,17^, to the best of our knowledge, there is no real-time classification system using 1EEG for mice achieving sensitivity and specificity for REM as high as the UTSN-L. Even with 1EEG, the UTSN-L achieved high-quality classification with a relatively small set of training data compared with previous networks (for example, ^7^), suggesting the robustness of the architecture.

One general difficulty in training systems for classifying sleep stages is that the amount of each sleep stage is imbalanced, with REM being particularly rare. In the future, this problem could be alleviated by using a loss function tailored for imbalanced data^18,19^. Another possibility is the use of an attention mechanism^20^. Often in sleep stage classification, human experts deliberately choose which part of the raw signal to focus on after observing the overall signal shape in an epoch. For example, if the overall amplitude of a signal is relatively small and consistent, human experts assume it could be REM and look for additional indicators (e.g., theta oscillation) to support their conclusion. In the future, an attention mechanism may be able to mimic such strategies.

In summary, we developed automatic, real-time sleep stage classification systems for mice using 1EEG input that provides high REM sensitivity and accuracy. These systems could potentially be used to analyze other time-series datasets. As they allow large-scale closed feedback loop applications, they can be employed to further investigate the functions of sleep.

## Methods

### Animals

All animal experiments were approved by the University of Tsukuba Institutional Animal Care and Use Committee. C57Bl6/J mice, which do not exhibit sleep–wake abnormalities, were maintained in a home cage (cylindrical Plexiglas cage: 21.9-cm diameter, 31.6-cm height) in an insulated chamber (45.7 × 50.8 × 85.4 cm), which was maintained at an ambient temperature of 23.5 ± 2.0 °C under a 12-h light/dark cycle (9 AM to 9 PM) with ad libitum access to food and water in accordance with institutional guidelines. The study was carried out in compliance with the ARRIVE guidelines.

### Implantation of EEG and EMG electrodes

At nine weeks of age, mice were anesthetized with isoflurane and fixed in a stereotaxic frame (Stoelting, USA). The height of bregma and lambda were adjusted to be equal. EEG/EMG electrodes were placed as previously described^3^. Briefly, EEG electrodes were stainless steel recording screws (Biotex Inc.) implanted epidurally at AP + 1.5 mm and − 3 mm and ML − 1.5 mm and − 1.7 mm, respectively, and EMG electrodes were stainless steel Teflon-coated wires (AS633, Cooner Wire, USA) bilaterally placed into the trapezius muscles.

### EEG and EMG recording

One week after electrode placement, mice underwent EEG and EMG recording in their home cage equipped with a data acquisition system capable of simultaneous video recording (Vital recorder, KISSEI COMTEC, Japan). Signals were recorded during ZT =  ~ 2 to 8 (~ 11 AM to 6 PM) for each mouse. Data were collected at a sampling rate of 128 Hz. Electric slip rings (Biotex Inc.) allowed mice to move and sleep naturally.

### Sleep stage analysis by human experts

Sleep stage analysis by human experts was conducted based on visual characteristics of EEG and EMG waveforms with the help of FFT and video surveillance of mouse movement. The EEG dataset consisted of 214 recordings in total. In most cases, two recordings on two consecutive days were obtained from each mouse. Recordings were divided into 10-s epochs. FFT analysis was performed using Sleep Sign software (KISSEI COMTEC).

Wakefulness was defined by continuous mouse movement or de-synchronized low-amplitude EEG with tonic EMG activity. NREM was defined by dominant high-amplitude, low-frequency delta waves (1–4 Hz) accompanied by less EMG activity than that observed during wakefulness. REM was defined as dominant theta rhythm (6–9 Hz) and the absence of tonic muscle activity. If a 10-s epoch contained more than one sleep stage (i.e., NREM, REM, or wakefulness), the most represented stage was assigned for the epoch. At least two experts agreed on the classification of each recording.

### Training, validation, and testing of UTSN and UTSN-L

To prepare the data for training, we normalized EEG data using the mean and standard deviation of all sample points in that recording. Let $x_{\tau }$ be the observed value of EEG at a sample time point $\tau$. In our experiment, the sampling frequency of EEG was 128 Hz, so *t* increments by 1/128 s. For validation and testing, we normalized EEG at each sample point (i.e., every 1/128 s) using the mean and standard deviation of all preceding values. In other words, for each sample point, the value was normalized as

$$\tilde{x}_{\tau } = \left( {x_{\tau } - \mu_{\tau } } \right)/\sigma_{\tau }$$

where

$$\mu_{\tau } = \frac{1}{\tau }\mathop \sum \limits_{s = 1}^{\tau } x_{s} \;{\text{and}}\;\sigma_{\tau }^{2} = \frac{1}{\tau }\mathop \sum \limits_{s = 1}^{\tau } \left( {x_{s} - \mu_{\tau } } \right)^{2} .$$

This process assimilates the real-time classification condition. On average, each recording consisted of ~ 2161 epochs.

### Hyperparameters

The network structure and hyperparameters of the CNN and LSTM in the UTSN and UTSN-L were selected from various possible models through preliminary evaluation (Suppl. Tables 2–3).

### Neural network architecture

A one-dimensional convolutional neural network processes the raw EEG data.

As the sampling rate is 128 Hz and the time window is 10 s, the segment is a 1280-dimensional vector. This network consists of stacked convolution layers that aggregate values of spatially neighboring nodes and output a lower-dimensional representation. Each convolution layer consists of one or more kernels (or filters) that act as local feature detectors. Let ${\varvec{z}}$ be the input vector to a $d$-diensional kernel ${\varvec{\kappa}}$. The output of a convolution layer is a vector ${\varvec{a}}$ whose components are obtained by $a_{\tau } = \mathop \sum \nolimits_{s = 1}^{d} \kappa_{s} z_{\tau - d + s}$.

### Model evaluation

Models were evaluated by criteria commonly used for multiclass and binary classification tasks (Suppl. Table 4).

### Statistical analysis

Statistical analysis was performed using GraphPad Prism version 7.04 for Windows (GraphPad Software, USA). Type I error was set at 0.05. Shapiro–Wilk tests were performed to assess the normality of data. Brown-Forsythe tests were performed to assess homogeneity of variance.

### Software implementation

We implemented the system using PyTorch. The neural network models were trained using NVIDIA Quadro RTX 8000 with 48 GB memory.

## Supplementary Information

Supplementary Figures and Tables.

Supplementary Video 1.

Supplementary Captions.

## Data Availability

Data underlying the results described in this manuscript are available at https://data.mendeley.com/drafts/5wtxz793my (for reviewing only). https://doi.org/10.17632/5wtxz793my.1 (after acceptance) except for the raw EEG and EMG recording data with stage labels, which are available at https://drive.google.com/drive/folders/1d27rv1bl9OT2X2KZ98LTXgtZO7AX8PBh?usp=sharing.

## Code availability

Code for UTSN and UTSN-L are available at https://github.com/tarotez/sleepstages.

## Materials availability

No special materials were created in this study.
