# Supplementary material for: Real-time, automatic, open-source﻿﻿ sleep stage classification system using single EEG for mice
Source: Sci Rep. 2021 May 27;11:11151. doi: 10.1038/s41598-021-90332-1 (PMC8160151; doi:10.1038/s41598-021-90332-1)
Supplement: Supplementary file 1 — Supplementary Figures and Tables. [file 41598_2021_90332_MOESM1_ESM.pdf]

Open-source, automatic, real-time sleep stage classification system using single EEG for mice

Taro Tezuka, Deependra Kumar, Sima Singh,
Iyo Koyanagi, Toshie Naoi, Masanori Sakaguchi

Suppl. Fig. 1. Network structure using the short-time Fourier transform (STFT).

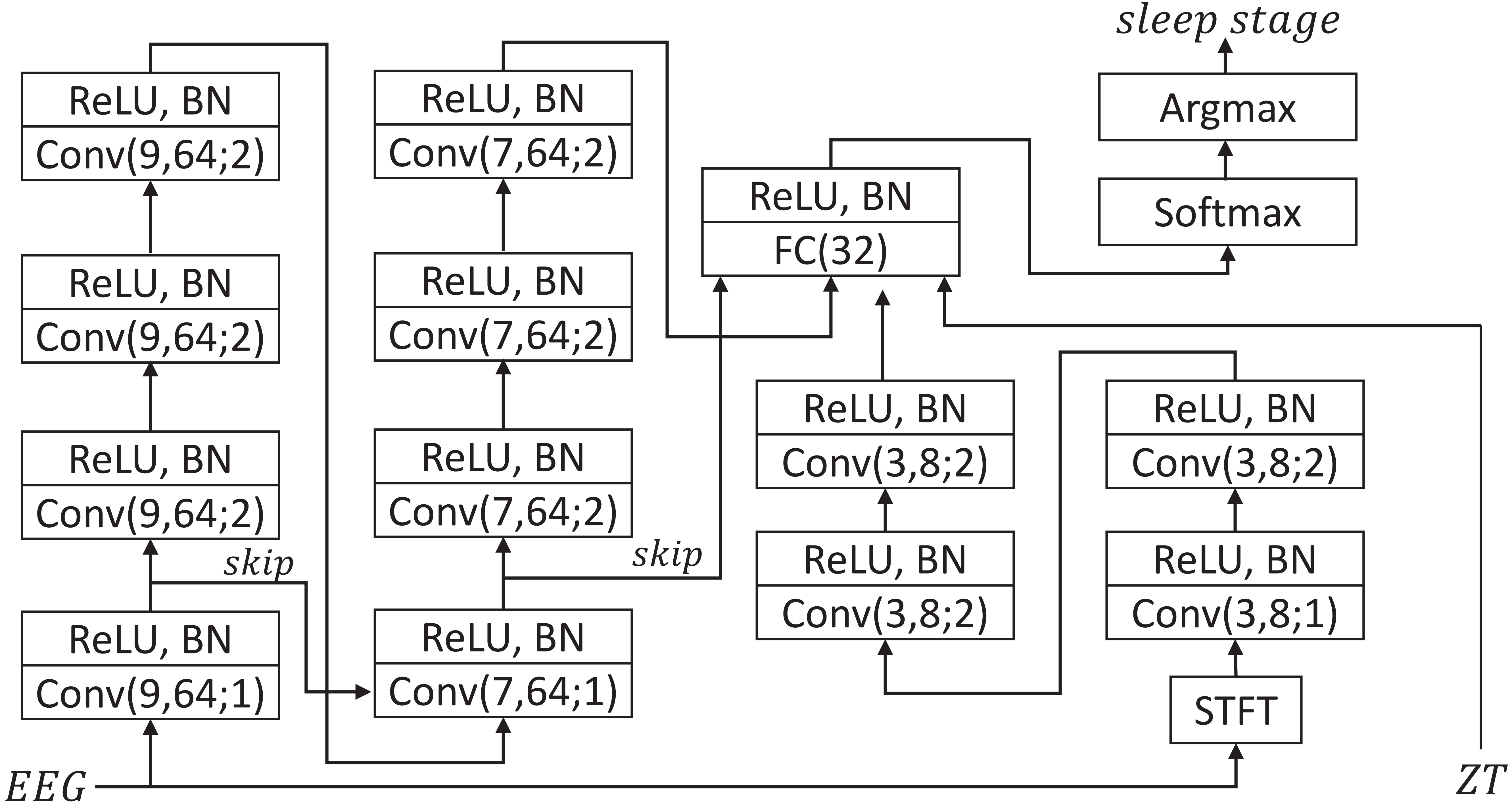

Suppl. Fig. 2. Network structure using short-time Fourier transform (STFT) and LSTM.

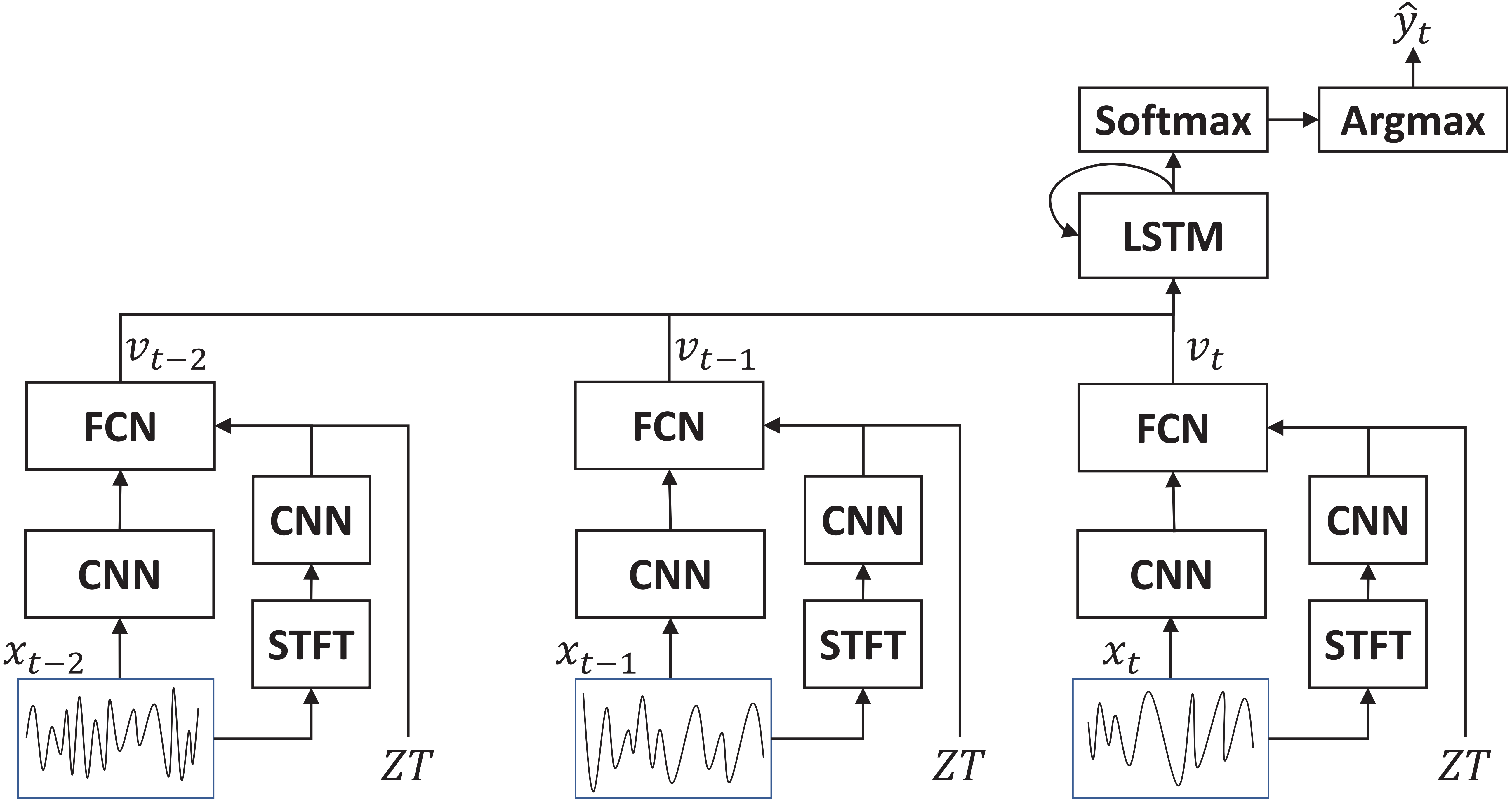

Suppl. Fig. 3. Performance comparison of the Fourier transform by cross-validation.

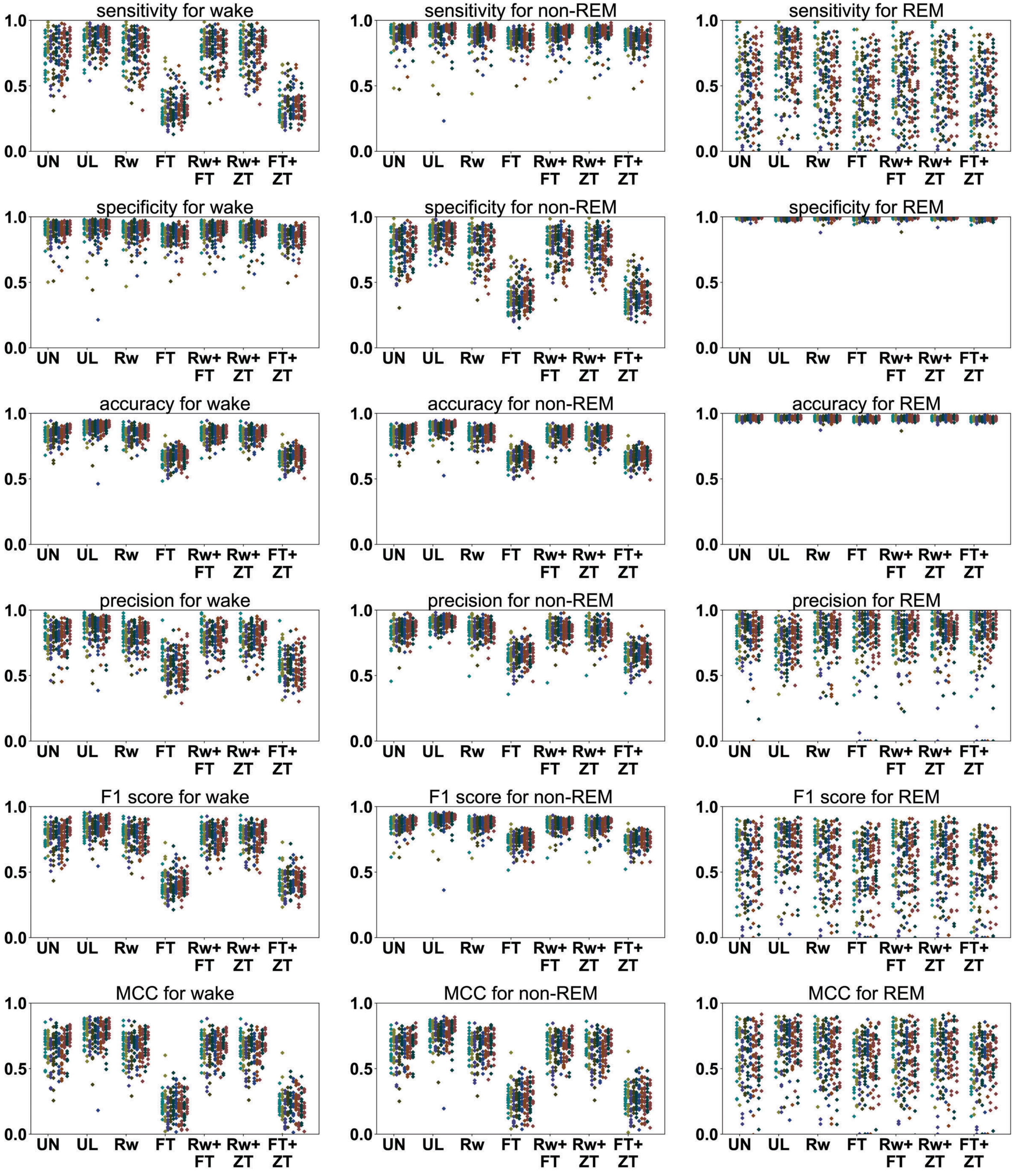

Suppl. Fig. 4. Performance comparison of the STFT by cross-validation.

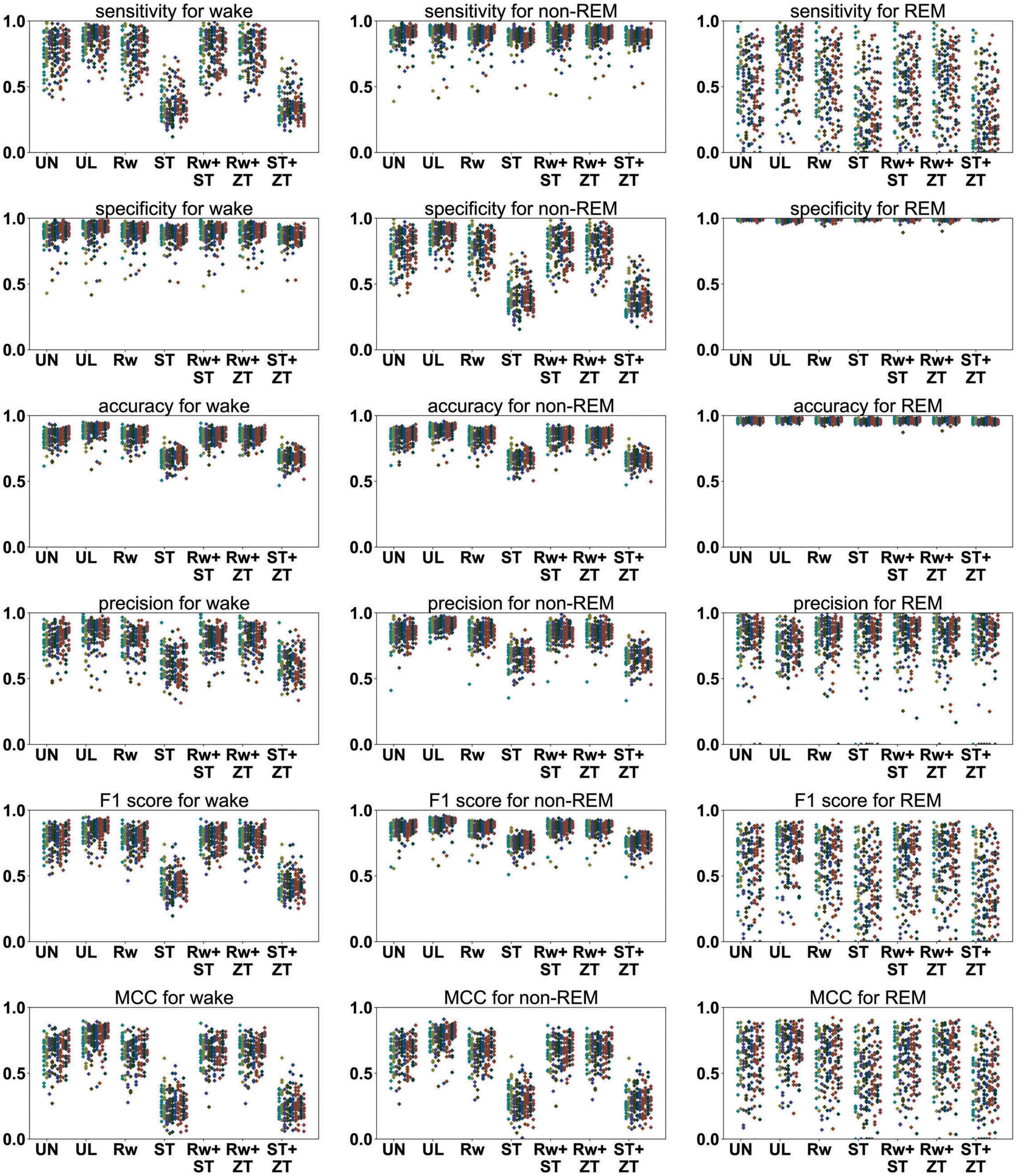

Suppl. Fig. 5. Comparison of sensitivity, specificity, accuracy, precision, F1 score, and MCC for different network models.

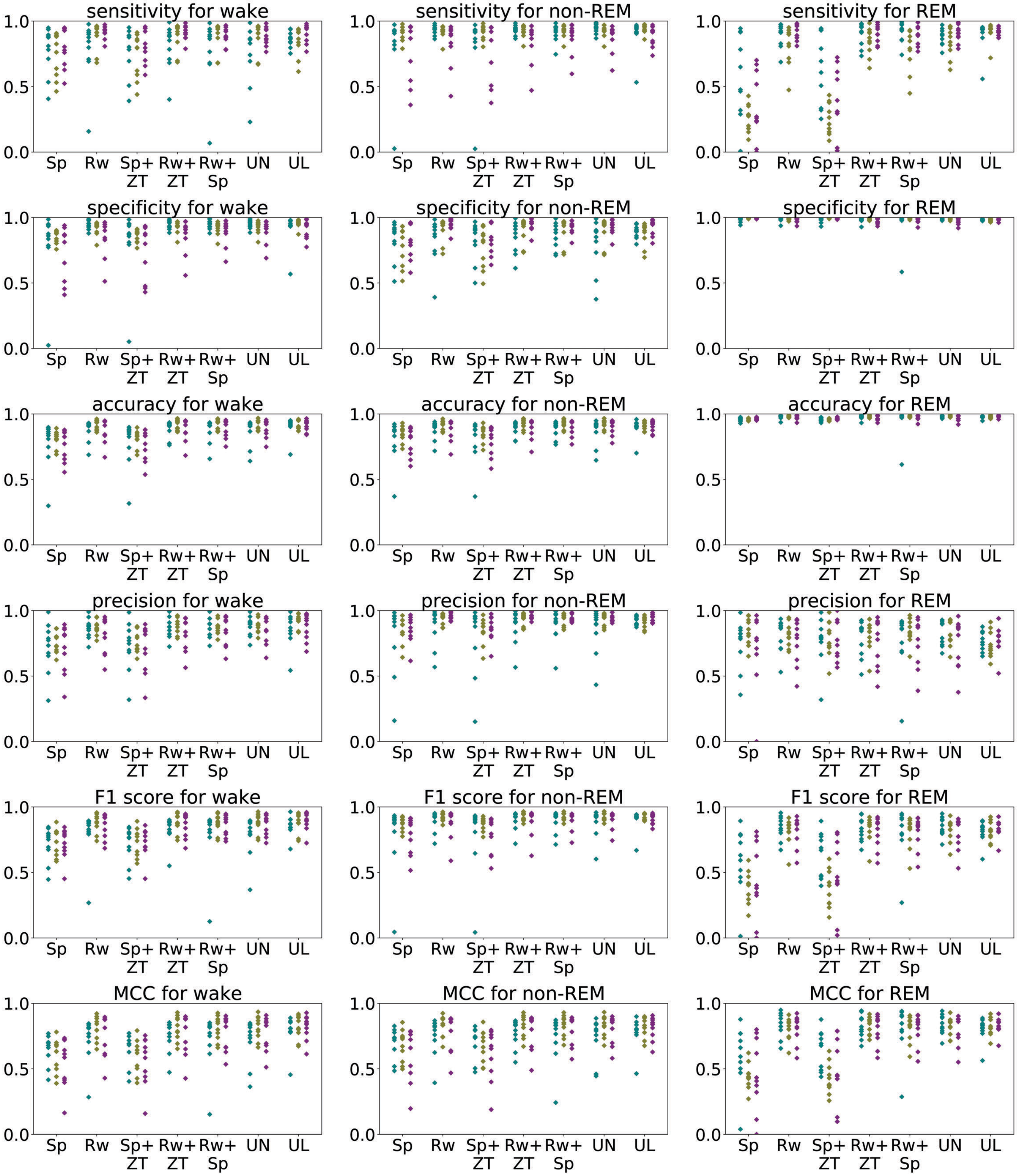

Suppl. Fig. 6

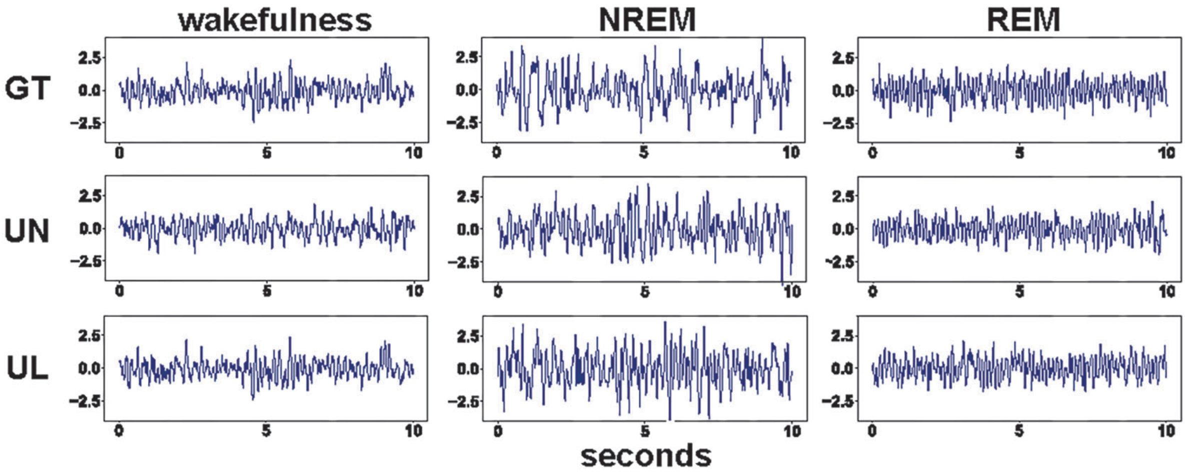

Suppl. Table 1. Comparisons between validations and tests.

Stage	measure	UTSN		UTSN-L	
		Validation	Test	Validation	Test
All	ACC	88.1	90.4	89.5	91.8
	mMCC	78.6	81.7	80.7	84.8
W	sensitivity	83.6	84.9	87.1	88.9
	specificity	92.4	95.0	92.9	95.2
	accuracy	89.0	91.3	91.1	92.9
	precision	87.0	89.6	88.6	91.4
	F1 score	84.0	86.9	87.3	89.9
	MCC	76.7	80.6	80.6	84.6
N	sensitivity	91.4	95.2	90.6	93.9
	specificity	87.4	88.2	89.7	91.6
	accuracy	89.4	92.1	90.4	92.8
	precision	90.9	91.6	92.5	93.8
	F1 score	90.4	93.3	91.2	93.7
	MCC	78.8	83.1	80.2	85.2
R	sensitivity	87.4	78.1	90.8	89.2
	specificity	98.3	98.6	98.1	98.3
	accuracy	97.7	97.4	97.6	97.8
	precision	79.2	82.5	75.2	78.6
	F1 score	82.3	78.5	81.9	82.9
	MCC	81.9	78.0	81.4	82.2

Suppl. Table 2. Network structures.

CNN	Conv(9,64;1) - Conv(9,64;2) - Conv(9,64;2) - Conv(9,64;2) - Conv(7,64;2) - Conv(7,64;2) - Conv(7,64;2) - Conv(7,64;2) - FC(32)
LSTM	Layer(32) - Layer(32) - Layer(32) - Layer(32) - Layer(32) - Softmax(10)

Suppl. Table 3. Hyperparameters for training the network.

Optimizer	Learning Rate	Batch Size	Epochs	Dropout Rate
Adam	0.0001	32	200	0.25

Suppl. Table 4. Evaluation criteria.

Overall accuracy (ACC)	$\frac{\sum_i C_{ii}}{\sum_i \sum_j C_{ij}}$
Overall multiclass MCC (mMCC)	$\frac{\sum_i \sum_j \sum_k (C_{ii}C_{jk} - C_{ij}C_{ki})}{\sqrt{\sum_i (\sum_j C_{ij}) (\sum_{h\|h\neq i} \sum_\ell C_{h\ell})} \sqrt{\sum_i (\sum_j C_{ji}) (\sum_{h\|h\neq i} \sum_\ell C_{\ell h})}}$
Sensitivity	$\frac{TP}{TP + FN}$
Specificity	$\frac{TN}{TN + FP}$
Accuracy	$\frac{TP + TN}{TP + TN + FP + FN}$
Precision	$\frac{TP}{TP + FP}$
F1 score	$\frac{2TP}{2TP + FP + FN}$
MCC	$\frac{TP \cdot TN - FP \cdot FN}{\sqrt{(TP + FP)(TP + FN)(TN + FP)(TN + FN)}}$

Suppl. Table 5. Ablation study for the Fourier transform by cross-validation.

Stage	measure	UTSN	UTSN-L	Raw	FT	Raw+FT	Raw+ZT	FT+ZT
All	ACC	82.3	**87.1**	82.5	64.2	82.2	81.7	64.2
	mMCC	66.4	**76.0**	66.8	29.3	66.2	65.2	29.3
W	sensitivity	76.3	**84.7**	77.0	33.4	77.0	75.6	34.9
	specificity	88.7	**90.8**	88.5	85.8	88.3	88.2	84.9
	accuracy	84.3	**88.8**	84.5	66.5	84.3	83.8	66.6
	precision	80.0	**85.2**	80.0	57.7	79.5	79.1	57.3
	F1 score	77.1	**84.3**	77.5	41.1	77.3	76.3	42.2
	MCC	65.8	**76.0**	66.2	21.9	65.7	64.4	22.3
N	sensitivity	89.3	**90.2**	88.8	86.2	88.8	88.5	85.2
	specificity	76.6	**86.3**	77.6	39.5	77.1	76.0	40.7
	accuracy	84.0	**88.7**	84.1	66.0	83.9	83.3	66.0
	precision	84.3	**90.3**	84.8	65.6	84.4	83.8	65.8
	F1 score	86.3	**89.9**	86.3	74.1	86.2	85.6	73.8
	MCC	66.4	**76.6**	66.8	26.7	66.2	65.0	26.7
R	sensitivity	49.9	**67.6**	52.4	44.0	48.8	51.6	43.9
	specificity	**99.3**	98.6	99.1	**99.3**	**99.3**	**99.3**	**99.3**
	accuracy	96.4	**96.8**	96.4	96.0	96.3	96.4	96.0
	precision	**81.4**	75.4	80.3	80.9	81.3	81.3	**81.4**
	F1 score	58.4	**69.0**	60.0	52.8	57.4	59.8	52.7
	MCC	59.9	**68.4**	61.0	54.6	58.9	61.1	54.6

Suppl. Table 6. Ablation study for STFT by cross-validation.

Stage	measure	UTSN	UTSN-L	Raw	ST	Raw+ST	Raw+ZT	ST+ZT
All	ACC	82.1	**87.2**	82.2	65.5	82.5	82.5	65.1
	mMCC	66.0	**76.1**	66.1	31.3	66.9	66.7	30.5
W	sensitivity	76.6	**85.1**	76.7	35.8	77.6	77.1	35.0
	specificity	88.4	**90.6**	88.3	86.7	88.3	88.4	86.9
	accuracy	84.3	**88.8**	84.2	68.0	84.6	84.5	67.9
	precision	79.8	**84.9**	79.5	60.6	79.9	79.6	60.6
	F1 score	77.2	**84.3**	77.2	43.8	77.8	77.4	43.2
	MCC	65.7	**76.0**	65.5	25.5	66.5	66.0	25.0
N	sensitivity	88.7	**90.0**	88.6	87.8	88.8	88.8	88.0
	specificity	76.5	**86.7**	77.0	40.3	77.7	77.6	39.1
	accuracy	83.6	**88.7**	83.7	67.3	84.2	84.1	66.9
	precision	84.2	**90.5**	84.3	66.4	84.9	84.8	66.0
	F1 score	85.9	**89.9**	86.0	75.2	86.3	86.3	75.0
	MCC	65.8	**76.6**	65.9	29.5	67.0	66.7	28.8
R	sensitivity	50.2	**68.7**	51.1	33.9	49.8	52.1	31.7
	specificity	99.3	98.6	99.2	99.6	99.3	99.2	**99.6**
	accuracy	96.4	**96.8**	96.4	95.6	96.3	96.4	95.5
	precision	81.1	75.8	80.9	79.1	**81.6**	80.7	80.9
	F1 score	58.8	**70.0**	59.3	43.2	58.4	60.0	41.2
	MCC	60.1	**69.4**	60.5	46.6	59.9	61.2	45.1

Suppl. Table 7. Performance comparison of the Fourier transform and STFT by cross-validation.

Stage	measure	UTSN		UTSN-L	
		FT	STFT	FT	STFT
All	ACC	82.3	82.1	87.1	**87.2**
	mMCC	66.4	66.0	76.0	**76.1**
W	sensitivity	76.3	76.6	84.7	**85.1**
	specificity	88.7	88.4	**90.8**	90.6
	accuracy	84.3	84.3	**88.8**	**88.8**
	precision	80.0	79.8	**85.2**	84.9
	F1 score	77.1	77.2	**84.3**	**84.3**
	MCC	65.8	65.7	**76.0**	**76.0**
N	sensitivity	89.3	88.7	**90.2**	90.0
	specificity	76.6	76.5	86.3	**86.7**
	accuracy	84.0	83.6	**88.7**	**88.7**
	precision	84.3	84.2	90.3	**90.5**
	F1 score	86.3	85.9	**89.9**	**89.9**
	MCC	66.4	65.8	**76.6**	**76.6**
R	sensitivity	49.9	50.2	67.6	**68.7**
	specificity	**99.3**	**99.3**	98.6	98.6
	accuracy	96.4	96.4	**96.8**	**96.8**
	precision	**81.4**	81.1	75.4	75.8
	F1 score	58.4	58.8	69.0	**70.0**
	MCC	59.9	60.1	68.4	**69.4**
