# Supplementary material for: Real-time, automatic, open-source﻿﻿ sleep stage classification system using single EEG for mice
Source: Sci Rep. 2021 May 27;11:11151. doi: 10.1038/s41598-021-90332-1 (PMC8160151; doi:10.1038/s41598-021-90332-1)
Supplement: Supplementary file 3 — Supplementary Captions. [file 41598_2021_90332_MOESM3_ESM.docx]

**Suppl. Fig. 1. Network structure using STFT.** The 1-12 Hz range of the STFT spectrum, which covers the critical characteristic oscillatory activity in delta (1-4 Hz) and theta (6-9 Hz) bands, was aggregated into 10 time bins and 22 frequency bins (i.e., 0.5 Hz per bin) and put into a CNN as a 10 × 22 matrix. The 2D-CNN transforms it into a lower-dimensional matrix, which is then flattened and concatenated with the raw signal and ZT.

**Suppl. Fig. 2. Network structure using STFT and LSTM.** ReLU, rectified linear unit; BN, batch normalization; Conv: convolution layer.

**Suppl. Fig. 3. Performance comparison of the Fourier transform by cross-validation.** The data consisting of 192 recordings were split into 10-folds. The result for each test recording is represented by a dot. Each color represents a fold. The networks were trained using hyperparameters indicated in Suppl. Table 3 except the number of epochs was set to 20. Rw, raw EEG; FT, Fourier transform; UN, UTSN; UL, UTSN-L. .

**Suppl. Fig. 4. Performance comparison of STFT by cross-validation.** Data consisting of 192 recordings were split into 10-folds. The result of each test recording is represented by a dot, and each color represents a fold. Networks were trained using hyperparameters indicated in Suppl. Table 3, except the number of epochs was set to 20. Rw, raw EEG; ST: STFT; UN, UTSN; UL, UTSN-L.

**Suppl. Fig. 5. Comparison of sensitivity, specificity, accuracy, precision, F1 score, and MCC for different network models.** Each training session (color-coded) contains 10 test results (indicated as 10 points). Sp, spectrum; Rw, raw EEG; Sp+ZT, FFT and ZT; Rw+ZT, FFT and ZT; Rw+Sp, raw EEG and FFT; UN, UTSN; UL, UTSN-L.

**Suppl. Fig. 6. Representative waveforms from testing***.* GT, ground truth; UN, UTSN; UL, UTSN-L.

**Suppl. Table 1. Comparisons between validations and tests.** W, wakefulness; N,

183 NREM; R, REM. Numbers indicate percentages (%).

**Suppl. Table 2. Network structures.** Conv($a$,$b$;$c$) indicates a block consisting of a one-dimensional convolution layer with kernel size $a$ having $b$ channels and stride set to $c$, followed by ReLU. FC($a$) is a block consisting of a fully connected layer having $a$ output channels followed by ReLU. We set the kernel size of the bottom layer to 9 to cover frequency components that signify sleep stages. We used a stacked bidirectional LSTM consisting of five layers. Layer($a$) is a layer of a stacked bidirectional LSTM having $a$ hidden nodes. The length of subsequences sent to the LSTM was set to 10 epochs, as average episode lengths range around 10 epochs (Fig. 3). All Conv($a$,$b$;$c$) and Dense($a$) blocks are batch normalized and use dropout. Layer($a$) also uses dropout.

**Suppl. Table 3. Hyperparameters for training the network.**

**Suppl. Table 4. Evaluation criteria.** Overall accuracy (ACC) and overall multiclass MCC (mMCC) are calculated as performance measures for multiclass classification. $C_{ij}$ is the $\left( i,j \right)$ element of the confusion matrix, where $i$ indicates a ground truth stage and $j$ indicates a predicted stage. The sum is taken over all stages (i.e., wakefulness, NREM, REM). Other criteria are calculated for each stage separately. For each target stage X, the multiclass classification results are relabeled into “X” or “not X”. For example, when the target stage is REM, labels after relabeling become “REM” or “not REM”. When the classifier labels an epoch as “REM”, the prediction is positive; when it labels an epoch as “not REM” (i.e., wakefulness or NREM), the prediction is negative. With these new labels, the performance measures are computed for the binary classification problem using *TP* (true positive), *FP* (false positive), *FN* (false negative), and *TN* (true negative).

**Suppl. Table 5. Ablation study for the Fourier transform by cross-validation.** Data consisting of 192 records were split into 10-folds. Networks were trained using hyperparameters indicated in Suppl. Table 3, except the number of epochs was set to 20. FT, Fourier transform; W, wakefulness; N, NREM; R, REM. Numbers indicate percentages (%).

**Suppl. Table 6. Ablation study for STFT by cross-validation.** Data consisting of 192 records were split into 10-folds. Networks were trained using hyperparameters indicated in Suppl. Table 3, except the number of epochs was set to 20. ST, STFT; W, wakefulness; N, NREM; R, REM. Numbers indicate percentages (%).

**Suppl. Table 7. Performance comparison of the Fourier transform and STFT by cross-validation.** Data consisting of 192 records were split into 10-folds. Networks were trained using hyperparameters indicated in Suppl. Table 3, except the number of epochs was set to 20. FT, Fourier transform; ST, STFT; W, wakefulness; N, NREM; R, REM. Numbers indicate percentages (%).

**Suppl. Video 1 Practical use of the sleep stage classification system.** This video explains how to install and use the sleep stage classification system. Link: https://drive.google.com/file/d/1BCpmgXcN5n4ioytv2xq0-TFfXZ0gEbW7/view?usp=sharing.
